# Processing figurative language: Evidence from native and non-native speakers of English

**DOI:** 10.3389/fpsyg.2022.1057662

**Published:** 2022-11-25

**Authors:** Reem Alkhammash

**Affiliations:** Department of English, Taif University, Taif, Saudi Arabia

**Keywords:** figurative language, metaphors, idioms, familiarity, transparency, comprehension, decomposability, non-native

## Abstract

In recent research on figurative phrases, factors (e.g., familiarity, transparency, meaning, and decomposability) have played a significant influence on how native and non-native English speakers (various L1 and L1 Arabic) acquire, process, and comprehend figurative language. These factors are not always described and operationalized precisely and are frequently considered autonomous. This study explores these factors in terms of language users’ ratings and their abilities to accurately infer meaning from a variety of familiar English and translated idioms and novel metaphors. A total of 123 participants from various language groups engaged in this study. The findings showed that familiarity is a strong predictor of transparency. In the ability to infer the meaning correctly, the best-fit model included an interaction between transparency and familiarity. The findings showed that guessing the meaning correctly led to a greater increase in the scores of transparency and decomposability. We explore how these factors work together to enable speakers to infer the meaning of both known and new figurative words at various levels. These results have significant implications for the learning and teaching of figurative phrases in the English as a foreign language (EFL) context, as they indicate variables that may make a figurative phrase valuable in terms of teaching time and effort.

## Introduction

Figurative language is broadly defined as any language in which a speaker means anything other than what is expressed literally and is a principal component of everyday communication (Dancygier and Sweetser, 2014). Native speakers use idioms, metaphors, similes, analogies, and other figures of speech so regularly that they appear commonplace. Yet, they constitute a significant barrier to second- and foreign-language learners. Numerous studies have pointed out the difficulty involved in using figurative phrases (FPs), as a major barrier among second-language learners from various L1 backgrounds (see, e.g., Cieślicka, 2015; Park, 2018; Chen, 2019). The difficulty associated with FPs in L2 English has been attributed to several sources. First, second-language learners have difficulty processing the mappings from the source or vehicle to the target or tenor. If we take the example of “Juliet is the sun,” we have Juliet as the tenor or target and the sun as the vehicle or source, and we can understand it to mean that there are mappings from the source to the target or that there is a ground with shared properties between the tenor and vehicle. Through mapping and/or the ground, we understand that Juliet glows and is warm and beautiful. A non-native speaker may encounter difficulties in understanding that Juliet is described as the sun because she has these outstanding physical or aesthetic qualities. Littlemore and Low (2006) attributed the difficulty in processing to a lack of “native speaker competence.” Second, FPs comprise two or more words that are viewed as a single unit, which is semantically understood thus (Johnson and Malgady, 1979). Such processing may cause learners to either misunderstand and/or not understand FPs. Littlemore et al. (2011) found that international students in British universities encountered over 40% of FPs that were challenging to understand in their lectures (Littlemore et al., 2011). Not all figurative language is created equal, given the different degrees of comprehensibility and processing for non-native speakers (Littlemore et al., 2011). Cacciari and Corradini (2015) noted that certain processing variations appear to exist across different forms of figurative expressions, in that fluent bilinguals appear to comprehend metaphors and irony (but not idioms) on the lines of monolinguals, but at a slower rate in general (Cacciari and Corradini, 2015). Metaphorical meanings are more accessible than conventional idiomatic ones, given that metaphors are classification assertions that are identical to those provided by literal language (Cacciari and Corradini, 2015). There is little research on FPs and the factors that determine L2 knowledge of the various senses. Only two recent studies (Carrol et al., 2018; Carrol and Littlemore, 2020) have examined this issue, to the best of my knowledge. Therefore, the current study examines whether factors like familiarity, transparency, meaning, and decomposability influence how native and non-native speakers of English comprehend the language figuratively. How do native L1 and L2 speakers of English vary in their capacity to comprehend and infer figurative meaning, and how does this connect to the broader set of skills that constitutes “figurative competence” (Pollio and Pollio, 1974, 200)?

## Literature review

### Brief accounts of metaphor processing

Metaphor theories differ in their assumptions about how metaphors are processed (Cacciari, 2015). Classical accounts of metaphors are a deviation from conventional (literal) language. The conventional pragmatic perspective holds that metaphors are utterances that are interpreted differently from their literal meanings. According to this approach, the literal interpretation should be sought and discarded until we arrive at the metaphorical interpretation. This means that inference is necessary to ascertain the intended message (Gibbs and Colston, 2012). Classical views consider metaphors “exceptional” patterns that exist in ordinary language and vary according to time and context. According to Grice (1967), a figurative interpretation is sought only after the literal reading is rejected. When one of the four maxims (e.g., the condition that an interpretation is informative) is broken, the literal interpretation is rejected. Figurative interpretation cannot begin until the complete utterance is processed (Grice, 1967).

In an alternative account, it was proposed that both metaphorical and literal interpretations are processed concurrently and employ the same processing mechanisms. Thus, processing interacts with contextual information for lexical elements and metaphorical language. Gibbs et al. (1997) demonstrated that metaphors need the same amount of processing time as literal phrases. When the context was appropriate, reaction times did not vary between figurative and literal expressions (Gibbs and Colston, 2012). However, Frisson and Pickering (2001) remarked that the reason for a typical difficulty in interpreting results from research on figurative language processing is due to confounding factors, such as plausibility, cloze likelihood, and word frequency, which are not usually controlled in several studies that compare processing durations for figurative and literal phrases. Such factors should be included as they can result in different conclusions around the potential difficulty of figurative language (Frisson and Pickering, 2001).

Blasko and Connine (1993, 13) defined familiarity as “the perceived experience with the metaphor.” They revealed that figurative interpretations of well-known metaphors were just as accessible as literal ones. They reported that figurative inferences of novel metaphors were more difficult to comprehend than literal interpretations. It is worth debating whether this measure of familiarity is truly aimed at the topic–vehicle relationship (for an overview of the topic–vehicle relationship, see Connor and Kogan, 2018). As Blasko and Connine proposed, a metaphor’s perceived experience may be influenced by earlier encounters with a group of related metaphors (e.g., from the same conceptual domains; Blasko and Connine, 1993). Thibodeau and Durgin (2011) proposed a potential alternative evaluation of metaphor familiarity and found a strong correlation between subjective assessments of familiarity [obtained by asking participants to rate a set of metaphors utilized in Jones and Estes (2005)] and metaphor frequency (obtained using Google as a corpus). They proposed that metaphor frequency can be used as an objective indicator of familiarity. They discovered a strong association between familiarity and aptness and did not find any relationship between conventionality and familiarity (Thibodeau and Durgin, 2011).

#### Familiarity

One factor that may affect metaphor processing is familiarity. Metaphors that make frequent use of close associates are more likely to gain familiarity with time. Associations may have evolved between these phrases because of speakers’ cumulative experience with a familiar metaphor (see, e.g., Bowdle and Gentner, 2005). Metaphors vary significantly in terms of familiarity, ranging from conventional or very familiar (babies are angels) to novel or highly unfamiliar (marriage is an alloy). Established metaphors do not activate the right hemisphere of the brain, whereas unfamiliar ones do (e.g., Mashal et al., 2005).

#### Transparency

Metaphor processing may be influenced by transparency, which refers to how easy it is to guess the meaning before it is known (Carrol et al., 2018). In comparison with an opaque phrase, readers can decipher the intended meaning more easily when it is easier to work out. For example, the FP expression “spell the bees” means “to reveal a secret” and is considered transparent (Keysar and Bly, 1999), whereas the FP expression “break the ice” is considered non-transparent (Smith, 2021). Skoufaki (2008) argued that idiom transparency intuition arises after learning and guessing an expression’s meaning and that contextual hints matter in constructing idiom transparency intuition.

#### Meaning

Processing or disambiguating figurative meaning aims to explain the implicit understanding of metaphor from a psycholinguistic perspective (see, e.g., Gibbs and Colston, 2012). According to Lakoff and MacCormac (1987), idioms that “make sense” are driven by an image and conceptual mapping. The motivating factors, according to Lakoff, make figurative phrases and their meanings comprehensible. Another classical example of a transparent figurative phrase is “to keep someone at arm’s length.” Lakoff argued that for many people, an image of an individual extending their arm would motivate their understating of the FP (Lakoff and MacCormac, 1987, 447–449). For metaphorical phrases that are not “understood” in the same way, learners appear to perform similarly in L1 and L2, indicating that it may be related to individual differences (Littlemore, 2001).

#### Decomposability

Certain figurative phrases were highly decomposable, with the meanings of their constituent pieces autonomously influencing their total figurative meanings. It relates to how closely metaphorical and literal interpretations correspond as independent variables. Other figurative phrases were impossible to decompose because it was difficult to see any connection between the phrase’s constituents and the idiom’s metaphorical meaning (Gibbs, 1994). There is a qualitative difference in processing decomposable versus non-decomposable figurative meaning (Caillies and Declercq, 2011). For example, it was found that after reading decomposable idiomatic phrases, figurative meanings were immediately activated, whereas it took longer for non-decomposable metaphoric figurative meanings to be activated. The finding supports the hypothesis that decomposable idiom comprehension takes place because of retrieving the figurative meaning from memory, but that non-decomposable metaphor comprehension requires a sense construction process (Caillies and Declercq, 2011).

### Native and non-native metaphor processing

The book, *Bilingual Figurative Language Processing*, was dedicated to non-native speakers’ figurative language processing. Section III of this book is most relevant to the current study. It discusses language processing in general and provides an overview of many known models of multilingual figurative language processing (García et al., 2015; Heredia and Cieślicka, 2015). Titone et al. (2015) provided an excellent summary of the present state of knowledge on bilingual idiom processing. They introduced the Constraint-Based Processing Model of L2, which postulates that bilinguals, like monolinguals, make simultaneous use of all information given (e.g., idiom familiarity or predictability) gathered from the direct recall and configurational analysis of idiomatic expressions during idiom comprehension (Titone et al., 2015). Cieślicka (2015) expanded on bilingual figurative language understanding and demonstrated how a bilingual processing model based on the literal analysis of L2 idioms (i.e., the Literal Salience Model) accounts for foreign-language learners’ processing of idiomatic phrases. Cieślicka covered several basic theories of L2 lexical acquisition in addition to evaluating a variety of characteristics (e.g., cross-linguistic resemblance, literal plausibility, and predictability), which influence idiom processing (e.g., Parasitic Hypothesis of vocabulary development; Cieślicka, 2015). Paulmann et al. (2015) analyzed phrasal verbs among native and non-native speakers using event-related potentials. Like idiomatic expressions, phrasal verbs (e.g., run into) are ambiguous and can be taken literally (e.g., to enter: He ran into the building) or metaphorically (e.g., to encounter someone: He ran into his old acquaintance). Paulmann et al. (2015) showed that proficient L2 English learners do not necessarily have difficulty comprehending phrasal verbs. Their overriding conclusion was that non-native, but fluent English speakers grasp phrasal verbs using processing mechanisms that are similar to those of native speakers (NS; Paulmann et al., 2015). Bromberek-Dyzman et al. (2015) presented an overview of the research on ironic processing and demonstrated that it is not defined by the literal/non-literal language difference in L1 and L2, but rather by the subjective connotation.

Research has investigated variables that affected metaphor processing (Gentner and Bowdle, 2001; Glucksberg and McGlone, 2001; Jankowiak et al., 2017; Kulkova and Fischer, 2019; Jankowiak, 2020; Khatin-Zadeh and Khoshsima, 2021). Carrol et al. (2018) investigated factors that have an important role in figurative processing among native and non-native speakers. As familiarity has a direct impact on transparency perception, semantic judgments cannot be considered independent. Although this has been mentioned in the literature before, it is not widely accepted in idiom research and has significant methodological implications. Transparency, decomposability, and motivation, for example, should be defined and operationalized more precisely. These judgments of transparency, decomposability, and motivation fluctuate significantly based on the stage at which they are made and may be best-considered interactions between a specific speaker and a specific phrase rather than as intrinsic idiom qualities. Carrol et al. (2018) state that once familiarity is considered, native and non-native speakers understand and determine the meaning of figurative terms in similar ways. When expressions are completely unfamiliar, their relative transparency impacts how well language users will be able to figure out what they imply. Inequalities are often attributable to native speakers’ larger vocabulary and cultural understanding. Many language learners may consider phrases fundamentally more transparent than would native speakers owing to their analytical approach. They argue that for L2 speakers, cross-linguistic influence has a clear impact on judgments and the capacity to recognize meaning.

Speakers view expressions in L2 that have the same words and meanings as idioms in the L1 as being more familiar (regardless of whether they have encountered the precise expression in the L2) and are more likely to identify the meaning correctly. They also think they are clearer and have native speakers’ competency in terms of being familiar with the idiomatic meaning and the ability to understand the meaning. This study relates to evidence that links L1 knowledge to multiword L2 processing in both online and offline language tasks. Future research may include some additional factors addressed here aside from continuing to deepen our understanding of how native speakers and language learners cope with and acquire idioms and figurative meaning in general (e.g., embodied simulation, cultural knowledge, and emotional engagement). Carrol and Littlemore (2020) looked at how native speakers understand novel figurative language in two eye-tracking studies. In Study 1, they established that known idiom (i.e., where meaning is provided) had a distinct advantage over unfamiliar idioms, but did not find a causal relationship between figurative and literal versions of conventional metaphors when compared to literal paraphrases of the same meaning. For L2 idioms, familiarity, transparency, and decomposability demonstrated facilitative effects. They found that the processing of idioms was affected by variables, such as literal plausibility and the dominance of metaphorical versus literal meanings. In Study 2, readers encountered familiar and unfamiliar idioms (or paraphrases) in contexts that either supported the meaning or were neutral. Supporting contexts had no effect on reading habits for either type of idiom or had no effect when readers were asked to determine the meaning later. Context can help with sense selection, but not when it comes to generating new senses, when factors like transparency gain relevance.

## Materials and methods

### Participants

A total of 123 participants engaged voluntarily in this study. The researcher adhered to the American Psychological Association (APA’s ethical guidelines), which prioritizes privacy, consent, and the ability to quit the task at any point. Three groups were selected. The first group comprised Arabic native speakers (L1 Arabic, *n* = 54, 43.9%). Their average age was 18.8 years (standard deviation SD = 1.7 years). They were all female undergraduate students who had enrolled at a Saudi University. They studied English for a mean of 6.8 years (standard deviation SD = 3.12 years) and lived in an English-speaking country for an average of 0.8 years (standard deviation SD = 2.62 years). The second group comprised English native speakers (*n* = 25, 20.3%) with an average age of 25.6 years (standard deviation SD = 2.2 years). Of these, 16 (38.1%) were female participants and 26 (61.9%) were male participants; 37 (88.1%) were undergraduate students, and five (11.9%) were postgraduate students. The third group comprised 43 “general” non-native second-language learners of English. They included 25 native Urdu speakers (20.3%) with a mean age of 21.6 years (standard deviation SD = 1.9 years), 14 native Hindi speakers (10.6%) with a mean age of 19.7 years (standard deviation SD = 178 years), and only three Filipino speakers (2.4%) with a mean age of 19.3 years (SD = 1.8 years). Of the total, 11 (44%) were female participants and 15 (56%) were male participants; 17 (68%) were undergraduate students, and 8 (32%) were postgraduate students. The non-native speakers’ group had studied English for an average of 14.1 years (standard deviation SD = 5.78 years) and lived in an English-speaking country for an average of 1.2 years (standard deviation SD = 2.71 years).

The L2 English and L1 Arabic speakers alone were asked to complete a vocabulary test. They were presented with 30 items. A modified version of the vocabulary size test was used to measure their proficiency (Nation and Beglar, 2007). The non-native speakers (NNS) scored higher (mean score = 18.9/30, SD = 9.39) than the L1 Arabic group (mean score = 13.4, SD = 7.00). In the self-rated reported usage of the English survey, listening skills were rated higher by the NNS group (mean listening score = 8.4, SD = 2.72) than the L1 Arabic group (mean listening score = 6.8, SD = 2.51). The NNS group rated reading higher (mean reading score = 8.4, SD = 3.27) than the L1 Arabic group (mean reading score = 7.1, SD = 2.40). The NNS group rated themselves higher for writing (mean writing score = 7.8, SD = 2.92) than the L1 Arabic group (mean writing score = 6.0, SD = 2.42). The NNS and L1 Arabic groups had mean speaking scores of 8 (SD = 2.88) and 5.7 (SD = 2.88), respectively. Regarding the self-rating of perceived proficiency in the usage of English by participants, they were asked to rate each of the ten dimensions out of five, and the NNS group rated themselves higher (mean = 31.9/50, SD = 16.0) than the L1 Arabic group (mean = 23.1, SD = 15.2).

### Materials and procedures

The study selected three types of figurative phrases; the first set of English idioms were adopted from Carrol et al. (2018). I modified the phrases to meet the cultural expectations of Saudi Arabic speakers. The second set were novel metaphors adopted from Carrol et al. (2018), and their selection met two conditions: They had to be unfamiliar and transparent. The third set were translated idioms from other languages; one of the subsets had translated Arabic idiomatic expressions selected by the researcher and was verified by two linguists. In Carrol et al. (2018), plausible meanings were generated following three criteria: (a) The alternative meanings should be different from the correct one, (b) the correct meaning should not be a paraphrase of the phrase in question, and (c) all meanings (alternative and correct) should be possible interpretations of the intended figurative meaning. The researcher adopted a process that increased the likelihood of providing possible interpretations by asking three speakers of different L1 (Filipino, Dutch, and English) to provide other possible meanings. When the speakers provided correct answers, the Arabic idiom was eliminated. All idioms chosen had not been encountered, and their answers fed the options for incorrect meanings. The final list comprised 22 English idioms, 22 novel metaphors, 22 translated idioms from German and Bulgarian, and 22 Arabic idioms.

The participants were told that a phrase would be shown on the screen. All of them answered the following questions: “How familiar is this phrase?” (they had to circle their answer on a seven-point Likert scale ranging from 1 = not at all familiar to 7 = very familiar), and “How easily could you guess the meaning of the phrase?” (they had to circle the answer on a seven-point Likert scale ranging from 1 = not easily at all to 7 = very easily). Then, four possible meanings were presented. The participants were instructed to choose the correct meaning of the phrase. They were presented with the correct meaning of the phrase and were asked to indicate whether there was a connection between the phrase and its meaning (they had to circle the answer on a seven-point Likert scale, ranging from 1 = no connection to 7 = very clear connection). The responses to the four questions provided data on four factors, namely familiarity, transparency, meaning, and decomposability. Each participant viewed the 88 items. This exercise lasted around 30–40 min for native English speakers. Each group of L2 English learners and native Arabic speakers took approximately 45–60 min.

## Results

### Familiarity

**FIGURE 1:**
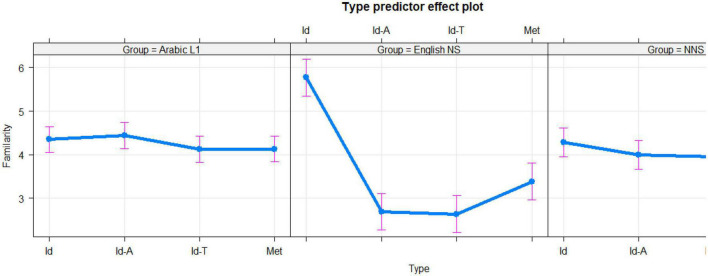
Ratings of familiarity broken down according to each phrase type (Id, English idioms; Met, novel metaphors; Id-T, generally translated idioms; and Id-A, Arabic idioms) for the Arabic L1 **(left panel)**, English native speakers (NS) **(middle panel)**, and non-native speakers (NNS) **(right panel)** groups. The 95% confidence interval is represented by error bars.

The English NS group was far more familiar with English idioms (score = 5.77) than the L1 Arabic (score = 4.35) and NNS (score = 4.28) groups. The difference between the English NS and L1 Arabic groups was statistically significant (*t* = 17.117, *p* < 0.00001). The difference between the English NS and L2 English NNS groups was statistically significant (*t* = 3.035, *p* < 0.00039). The L1 Arabic group was marginally better than the NNS group in terms of familiarity with English idioms (means of 4.35 and 4.28, respectively). However, the difference was not statistically significant (*t* = 0.8667, *p* = 0.3863). For a visual representation of familiarity ratings, see Figure 1 below.

However, in terms of novel metaphors, the L1 Arabic group (mean score = 4.13) was more familiar with novel metaphors than the English NS group (mean score = 3.39) with a significant margin (*t* = 7.978, *p* < 0.00001), but the L1 Arabic group’s rating of familiarity was not significantly different from that of the NNS group (mean score = 4.18; *t* = 0.623, *p* = 0.5335). The NNS group was more familiar with novel metaphors than the English NS group (mean scores of 4.18 and 3.39, respectively, *t* = 7.952, *p* < 0.00001). L1 knowledge influenced familiarity ratings for the translated Arabic idioms. The mean of the L1 Arabic group was 4.44, in terms of familiarity with Arabic idioms, substantially exceeding both the English NS (mean score = 2.69, *t* = 19.629, *p* < 0.00001) and NNS (mean score = 3.99, *t* = 5.775, *p* < 0.00001) groups. The English NS group was far less familiar with Arabic idioms when compared to the NNS group (*t* = 13.619, *p* < 0.00001). The L1 Arabic group’s mean score was 4.12 for familiarity with generally translated idioms, which was higher than that of both the English NS (mean score = 2.64, *t* = 16.521, *p* < 0.00001) and NNS (mean score = 3.96, *t* = 2.084, *p* = 0.03731) groups. The English NS group’s familiarity with generally translated idioms was lower than the NNS group (*t* = 13.748, *p* < 0.00001).

### Transparency

**FIGURE 2:**
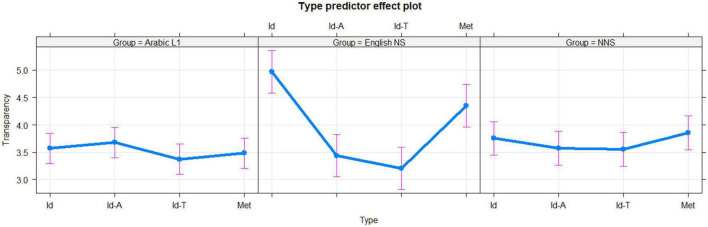
Ratings of transparency broken down according to each phrase type (Id, English idioms; Met, novel metaphors; Id-T, generally translated idioms; and Id-A, Arabic idioms) for the Arabic L1 **(left panel)**, English native speakers (NS) **(middle panel)**, and non-native speakers (NNS) **(right panel)** groups. The 95% confidence interval is represented by error bars.

For English idioms, the English NS group was more likely to rate English idioms as more transparent (mean = 4.97) than both the Arabic L1 group (mean = 3.57, *t* = 17.022, *p* < 0.00001) and the NNS group (mean = 3.76, *t* = 13.625, *p* < 0.00001). The difference in transparency scores for English idioms between the NNS (mean = 3.76) and Arabic L1 (mean = 3.57) groups was statistically significant (*t* = 2.3962, *p* = 0.01666). These results were consistent even after accounting for the effect of familiarity (*t* = 9.42644, *p* < 0.00001) for the difference between the Arabic L1 and English NS (*t* = 6.315629, *p* < 0.00001), Arabic L1 and NNS, and English NS and NNS (*t* = 2.767943, *p* = 2680.01745631) groups. For generally translated idioms, the Arabic L1 group scored a mean of 3.37, which exceeded that of the English NS group (score = 3.21). This difference was statistically significant (*t* = 2.0885, *P* = 0.03698) and improved upon accounting for the familiarity effect too (*t* = 6.00781, *p* < 0.00001). However, the Arabic L1 group scored the translated idioms as less transparent than the NNS group (mean score = 4.35, *t* = 2.3772, *P* = 0.01755; became *t* = 3.412871, *p* = 0.002411837) after accounting for familiarity. The NNS group outperformed the English NS group with a significant margin (*t* = 3.9664, *p* = 0.00008; after accounting for familiarity, it became *t* = 2.679873, *p* = 0.022161618). As for the transparency of translated Arabic idioms, the Arabic group score (mean = 3.67) was significantly higher than that of the English NS group (mean = 3.43, *t* = 3.1128, *p* = 0.001895), but not statistically different from that of the NNS group (mean = 3.58, *t* = 1.3721, *p* = 0.1702). The difference between the NNS and English NS groups was statistically insignificant (*t* = 1.6825, *p* = 0.0927). For a visual representation of transparency ratings, see Figure 2 above.

In relation to transparency for novel metaphors, the Arabic L1 group underperformed (mean = 3.48) when compared to both the English NS (mean score = 4.35, *t* = 10.735, *p* < 0.00001) and NNS (mean score = 3.85, *t* = 4.7853, *p* < 0.00001) groups. The English L1 group scored higher than the NNS group (*t* = 5.6874, *P* < 0.00001). All these measures stayed the same after accounting for the effect of familiarity (*t* = 20.7432, *p* < 0.00001; *t* = 4.498109, *P* = 0.000035; *t* = 9.582462, *p* < 0.00001, respectively).

The L1 Arabic group (mean score = 4.13) was more familiar with novel metaphors than the English NS group (mean score = 3.39) with a significant margin (*t* = 7.978, *p* < 247 0.00001), but the L1 Arabic group’s rating of familiarity was not significantly different when compared with the NNS group (mean score = 4.18; *t* = 0.623, *p* = 0.5335). The NNS group was more familiar with novel metaphors than the English NS group (mean scores 4.18 and 3.39, respectively, *t* = 7.952, *p* < 0.00001). L1 knowledge influenced familiarity ratings for the translated Arabic idioms. The L1 Arabic group scored a mean of 4.44 in terms of familiarity with Arabic idioms, substantially exceeding both the English NS (mean score = 2.69, *t* = 19.629, *p* < 0.00001) and NNS (mean score = 3.99, *t* = 5.775, *p* < 0.00001) groups. The English NS group was far less familiar with Arabic idioms than the NNS group (*t* = 13.619, *p* 254 < 0.00001). The L1 Arabic group’s mean score was 4.12 for familiarity with generally translated idioms, higher than both the English NS (mean score = 2.64, *t* = 16.521, *p* < 0.00001) and NNS (mean score = 257 3.96, *t* = 2.084, *p* = 0.03731) groups. Remarkably, the English NS group’s familiarity with generally translated idioms was lower than the NNS group (*t* = 13.748, *p* < 0.00001).

### Meaning

We alternated between mixed logistic regression models and started with an omnibus model to explore the fixed effects of group and type. We included familiarity and transparency to explore the extent of their contributions, and the upcoming models were sequentially compared. Both familiarity (chi-squared = 8.037, *p* = 0.004583) and transparency (chi-squared = 19.178, *p* = 0.000012) improved the model fit significantly when each of them was added to the omnibus model. The inclusion of both variables made a significant improvement in model fit when compared to the familiarity-only model (chi-squared = 11.974, *p* = 0.000539). However, when both variables were included, the fit was not substantially better than the transparency-only model (chi-squared = 0.8329, *p* = 0.3614).

**FIGURE 3:**
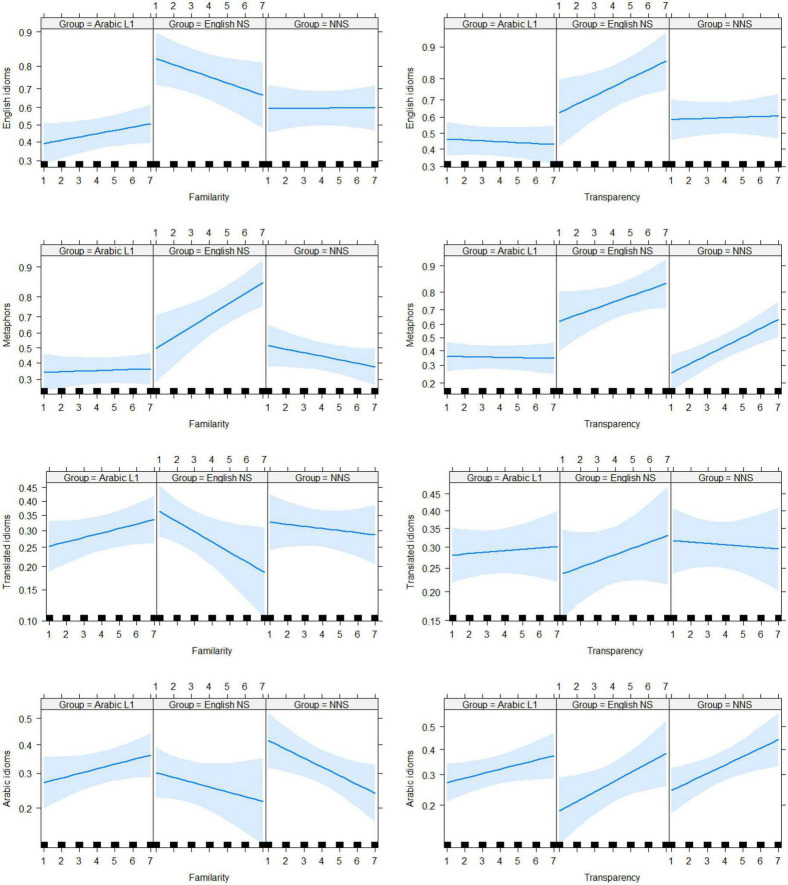
Logistic model estimates for the effects of both familiarity **(left)** and transparency ratings **(right)** broken down further according to phrase type: English idioms **(top row)**, metaphors **(second row)**, translated idioms **(third row)**, and Arabic idioms **(bottom row)**. Blue shading indicates 95% confidence intervals. The correct mean is expressed on the logit scale.

Both familiarity and transparency were positively associated with the meaning: for familiarity (mean score for those who identified the correct meaning = 4.21, mean in those who did not = 3.98, *t* = 6.1656, *p* < 0.00001); for transparency (mean score for those who identified the correct meaning = 3.95, mean in those who did not = 3.49, *t* = 13.577, *p* < 0.00001). To explore this further, we fit separate models for each phrase type. For each model, we added familiarity to see whether it improved the model and then added transparency to see whether it made any additional improvements. The variables were added in this order on the grounds that if a phrase is known, its meaning can be retrieved directly. Thus, relative transparency had an effect only for unknown phrases. Figure 3 shows the contribution of familiarity and transparency per speaker group for each phrase type.

For idioms, adding familiarity to the model with the fixed effect of groups substantially improved the model fit (chi-squared = 5.9773, *p* = 0.01449). The addition of transparency to the model with familiarity and group improved the fit (chi-squared = 7.6161, *p* = 0.005785). Including an interaction between group and familiarity improved the omnibus model further (chi-squared = 19.19, *p* = 0.00025). Transparency inclusion as a fixed effect improved the model (chi-squared = 8.0644, *p* = 0.004514) and as an interaction between transparency and group (chi-squared = 19.958, *p* = 0.0001732). The best-fitting model included familiarity and transparency in interacting with the group. For metaphors, the inclusion of familiarity (chi-squared = 3.1411, *p* = 0.3704) and transparency as a fixed term (chi-squared = 0.2503, *p* = 0.6168) and as an interaction term (chi-squared = 5.0146, *p* = 0.1707) in the model did not improve the model fit. This highlighted the absence of an impact on familiarity and transparency around identifying the correct meaning of metaphors. The best model, upon comparing all possible models, was the one that included interaction terms with both groups and transparency. In this model, the English NS group scored significantly better than the Arabic L1 group (*z* = 2.521, *p* = 0.0117), but the NNS group did not (*z* = 1,856, *p* = 0.0635). The effect of familiarity and transparency on getting the correct meaning of a metaphor was not statistically significant (*z* = 1.561, *p* = 0.1185, and *z* = −0.502, *p* = 0.6155, respectively).

For translated idioms, adding familiarity with group interaction term improved the model fit significantly (chi-squared = 7.8686, *p* = 0.04881). However, including a further transparency term did not help the model fit both as a fixed (chi-squared = 0.3009, *p* = 0.5833) and interaction term (chi-squared = 1.2284, *p* = 0.7462). The best-fit model was the one with an interaction term for familiarity with the group, in which the English NS group outperformed the Arabic L1 group, but the NNS group did not (*z* = 1.946, 325*p* = 0.05160). For Arabic idioms, including a familiarity term did not improve the model (chi-squared = 7.3418, *p* = 0.06176). However, including a fixed transparency term in the omnibus model resulted in substantial improvement (chi-squared = 11.329, *p* = 0.000763). This model improved through the inclusion of an additional familiarity interaction term (chi-squared = 8.3131, *p* = 0.03996), but did not improve upon adding a transparency interaction term (chi-squared = 1.4125, *p* = 0.4935). The best fit was for the model that included fixed transparency and interaction familiarity terms, where transparency exhibited an improved ability to get the correct meaning (*z* = 3.527, *p* = 0.000421), but familiarity did not (*z* = 1.258, *P* = 0.208250). The NNS group outperformed the Arabic L1 group (*z* = 2.435, *p* = 0.014887), but the English NS group did not (*z* = 0.580, *p* = 0.561727).

Logistic model estimates for the effects of both familiarity (left) and transparency (right) ratings were broken down further based on phrase type, as follows: English idioms (top row), metaphors (second row), translated idioms (third row), and Arabic idioms (bottom row). The blue shading indicates 95% confidence intervals. The correct mean is expressed on the logit scale.

### Decomposability

The transparency (i.e., the ratings of how easy it was to guess the meaning of a figurative phrase) was positively correlated with the subsequent decomposability scores (i.e., the clarity of connection between individual words and the overall correct meaning of the figurative phrase). The correlation was statistically significant (*r* = 0.4333678, *p* < 0.00001). However, transparency’s mean score was 3.681, marginally higher than the decomposability’s mean score of 3.679 (*t* = 0.082822, *p* = 0.934). Thus, decomposability ratings (after the meaning was known) were, in general, similar to transparency ratings (before the meaning was known).

**FIGURE 4:**
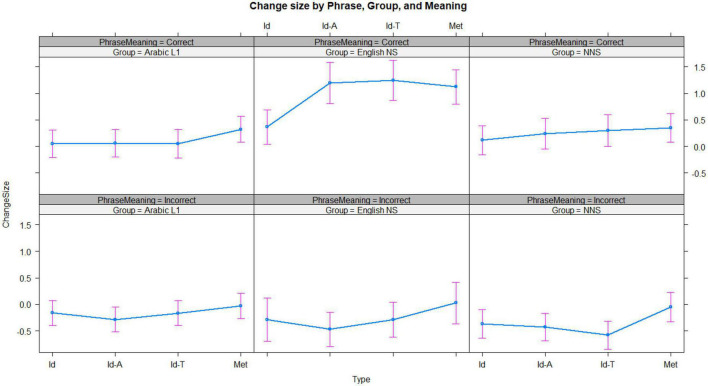
The size of change from initial transparency to subsequent decomposability scores for each phrase type, for items where the meaning was correctly identified **(top three panels)** and was not **(bottom three panels)**. Id, English idioms; Met, metaphors; Id-T, generally translated idioms; and Id-A, Arabic idioms. Error bars represent 95% confidence intervals.

A model with the size of the change from initial transparency to final decomposability as the dependent variable was constructed to evaluate the effect of different covariates. We first included the fixed effects of Type and Group. Then, initial transparency ratings were added, which significantly improved the model both as a fixed effect (chi-squared = 4476, *p* < 0.00001) and as part of the interaction with Type and Group (chi-squared = 112.66, *p* < 0.00001). Second, familiarity was included, which also added to the model fit both as a fixed effect (chi-squared = 387.08, *p* < 0.00001), and as part of an interaction with the Type and Group (chi-squared = 111.58, *p* < 0.00001). Familiarity significantly improved the size of the change from decomposability to transparency (*t* = 8.421, *p* < 0.00001). We finally checked whether adding meaning would make an additional improvement on the grounds that whether participants got the answer right or wrong may be important to how they re-evaluated their original rating for transparency. The model comparison showed that the inclusion of meaning made an improvement, both as a fixed factor (chi-squared = 493.35, *p* < 0.00001) and as part of an interaction with the group and type (chi-squared = 255.07, *p* < 0.00001), and when it was included, familiarity remained a significant predictor (*t* = 8.663, *p* < 0.00001). The best-fitting model included interactions among the Type, Group, and Familiarity; and Type, Group, and Transparency; and Type, Group, and Meaning. This model suggests that participants were more likely to show an increase from transparency to decomposability if they identified the correct meaning successfully, when compared to when they were incorrect. Figure 4 highlights patterns according to phrase type and speaker group, and according to whether participants identified the meaning of each phrase accurately. The findings presented in Figure 4 suggest that identifying the meaning of a phrase correctly led to a greater increase in the score from transparency to decomposability than if the meaning was not identified.

The final model confirmed that this difference was greater for the NNS group (effect = −0.6030869, *p* = 0.007630426). The English NS group did not differ substantially from the Arabic L1 group in this model (effect = 0.2193054, *p* = 0.4971379). To interpret the effects better, we fitted separate models for each phrase type. Each model included interactions among group, transparency, and meaning. For English idioms, all three groups showed a non-significant negative change from transparency to decomposability when they did not identify the correct meaning, with the only significant between-group difference being recorded between the NNS and Arabic L1 groups (*t* = 2.703, *p* = 0.00687). There was a significant effect of initial transparency (*t* = −24.791, *p* < 0.00001) and significant interaction between group and transparency for the NNS group (*t* = 2.354, *p* = 0.018573) and L1 Chinese (*t* = 4.79, *p* < 0.001). For all three groups, when the meaning was not identified correctly (therefore, presumably not known in the first place), subsequently learning the meaning made little difference to how decomposable the idioms were perceived to be (relative to the original rating for transparency). In contrast, identifying the meaning correctly (either because this was known or correctly inferred) led to a positive change, which was more pronounced for the NNS group when compared to the L1 Arabic speakers. For metaphors, there was a substantial effect for initial transparency scores on the size of change from transparency to decomposability (*t* = −26.653, *p* < 0.00001). There was a significant interaction between English NS and initial transparency score (*t* = 3.612, *p* = 0.000304), between English NS and getting the correct meaning (*t* = 5.551, *p* < 0.00001), and between transparency and correct meaning (*t* = 3.688, *p* = 0.000023).

For Arabic idioms, as expected, both English NS and NNS groups showed lower change size when compared to the Arabic L1 group (*t* = −2.054 and *p* = 0.039976, *t* = −2.651, and *p* = 0.008025, respectively). Initial transparency scores were highly associated with the size of change (*t* = −26.573, *p* < 0.00001), indicative of the fact that a lower initial transparency score would leave more room for change than a high initial score.

Guessing the correct meaning was associated with change size (*t* = −2.097, *p* = 0.0359936) as well. There was a significant interaction between transparency and the NNS group (*t* = 2.382, *p* = 0.0172189) and between correct meaning and both the English NS (*t* = 4.351, *p* = 000014) and NNS (*t* = 1.963, *p* = 399 0.049646) groups. For translated idioms, both the English NS and NNS groups showed a smaller change when compared to the Arabic L1 group (*t* = −3.443 and *p* = 0.000575, *t* = −5.375, and *p* < 0.00001, respectively). Initial transparency score and correct meaning individually indicated a smaller change (*t* = −28.787, *p* < 0.0001; *t* = −3.098, *p* = 0.0019483, respectively).

Transparency interacted significantly with both English NS and NNS groups (*t* = 3.086 and *p* = 0.0020287, *t* = 5.609, and *p* < 0.0001, respectively). Correct meaning interacted with both English NS and NNS groups (*t* = 4.951, *p* < 0.00001, and *t* = 2.929, *p* = 0.0034005, respectively) and with transparency (*t* = 4.548, *p* < 0.00001). The results suggest that initial transparency ratings had an inverse relationship with the size of change toward final decomposability scores. The ability to correctly identify the meaning of the phrase posed a negative relationship with the size of change in translated and Arabic idioms, but not in metaphors or English idioms.

## Discussion

We investigated the factors that influence the way native and NNS comprehend figurative language. The results show that there is a significant difference among the English NS, English NNS, and L1 Arabic groups. The English NS group was more familiar with English idioms than both the English NNS and L1 Arabic groups. Yet, L1 Arabic speakers were slightly more familiar with English idioms than NNS speakers. This study produced results that differed from those of Carrol et al. (2018). Both L1 Arabic and the English NNS groups had similar familiarity ratings to novel metaphors. However, significant differences were found between the English NS and L1 Arabic groups, but not between the English NS and NNS groups. The English NS group was the least familiar with novel metaphors. This is not in line with Carrol et al. (2018). In generally translated idioms, the L1 Arabic group was more familiar with them than the English NS group and the English NNS group. There was a statistical difference between the English NS and NNS groups, wherein the former’s familiarity with generally translated idioms had the least familiarity ratings. This study produced results that differed from those of Carrol et al. (2018), in terms of familiarity with generally translated idioms. Carrol et al. (2018) found that L1 Chinese speakers were more familiar with the translated idioms than the English NS and NNS groups and that there were no differences between the English NS and NNS groups, and L1 Chinese speakers. However, L1 Arabic speakers were more familiar with generally translated idioms. The finding on the familiarity ratings of Arabic idioms was similar to Carrol et al. (2018) in that L1 knowledge influenced familiarity ratings. With transparency ratings alone, no differences between groups were found in Carrol et al. (2018). The inclusion of familiarity as a covariate in a three-way interaction besides type and group proved to strengthen the findings of Carrol et al. (2018), especially for the English NS group. Both the English NNS and L1 Chinese groups found the English idioms to be more transparent than the English NS group. However, in this study, the transparency mean scores for the English NS group were higher than those of both the English NNS group and L1 Arabic speakers with the statistical difference between the English NS and NNS groups, and the English NS and L1 Arabic groups before including familiarity as a covariate. These findings were consistent even when familiarity was included. In this study, novel metaphors were more transparent to the English NS group, followed by the English NNS group, and then followed by L1 Arabic speakers before and after the inclusion of familiarity. This is inconsistent with the findings of Carrol et al. (2018), where novel metaphors were more transparent for the English NNS than NS groups and L1 Chinese speakers. Metaphors were considered more transparent by the NNS speakers, in the model both with and without familiarity as an interaction term.

When familiarity and transparency were introduced to the omnibus model, both improved models fit to each phrase type by the speaker group. The English NS group outperformed the Arabic L1 group significantly, whereas the NNS group did not. The model containing the interaction between language groups and familiarity was the best fit. In this model, the English NS group outperformed the Arabic L1 group, but not the NNS group. This is in line with Carrol et al. (2018) in that familiarity is a strong predictor of meaning and how well participants inferred the meaning correctly. The relationship between meaning and form is established and harder to ignore, familiar phrases appear more transparent, and unfamiliar words become less transparent. This indicates that accurately predicting the meaning increased the ratings of transparency and decomposability, on the lines of Carrol et al. (2018).

Finally, this study investigated how these variables interact to enable speakers to infer meaning at various levels from both known and novel figurative words. As familiarity affects perceptions of transparency and meaning, they cannot be considered autonomous. Cross-language influence affects L2 speakers’ perceptions and the ability to recognize meaning. L2 speakers view expressions in L2 that have the same words and meanings as idioms in L1, as more familiar. They see them as more transparent, just as native speakers infer the meaning in the idioms they know well. The ability to competently interpret idiomatic language is found in two critical areas: a semantic inferencing skill that helps derive meaning from analogy, analysis, and context, and a comprehensive knowledge of the conventional figurative phrases in the language (Gibbs, 1987, 1994; Gibbs et al., 1997; Miller et al., 2020).

The findings show that the results differ from Carrol et al. (2018). It is likely that language skills for L2 speakers have different trajectories of development given the learners’ individual differences. Even for native speakers, processing figurative language requires a range of linguistic, pragmatic, and cognitive skills to derive an appropriate interpretation (Carrol, 2021, 307). It is possible that these results may be inconsistent with those of Carrol et al. (2018), as cross-language influence may have had an effect on judgments and the ability to determine meaning for L2 speakers. When L2 speakers encounter FPs that are the same in the L1, they perceive them as more familiar and transparent and can easily identify their meanings, whereas if the FPs are not the same, the judgments will differ.

A significant contribution of this study is that its findings reveal certain difficulties that English as a foreign language (EFL) learners may face while processing FPs. First, as EFL learners in the present study found highly familiar FPs easy to recall and recognize, these should not be the focus of teaching interventions. Instead, language teachers may need to devote classroom time to senses that are not that transparent and cannot be decomposed as easily. If learners are expected to use FPs productively, then familiarity, transparency, and decomposability should also be driving forces for item selection. Thus, FP classroom instruction can be considered compensation for the scarcity of encounters available through natural language exposure. This is especially the case for the less transparent FPs senses, which were found difficult to recall/recognize by our EFL learners and those in the literature (see Martinez, 2013). One way for this is to use cognitive teaching approaches, such as metaphor awareness (see Boers, 2004). To the best of my knowledge, only very few studies (e.g., Yasuda, 2010; Strong and Boers, 2019) have explored the effectiveness of various teaching techniques on FP knowledge development. More research is needed in this area.

Although the NNS group’s proficiency is higher than that of the L2 Arabic native speakers, one could argue that one of the limitations of this study is that it does not identify within-group differences between the proficiency levels of different language groups, which requires a more comprehensive test (see Tremblay, 2011).

## Conclusion

Research on figurative phrases has shown that various factors (e.g., familiarity, transparency, meaning, and decomposability) impact how native and non-native English speakers comprehend figurative language. However, these variables are not often explicitly characterized and operationalized and are frequently considered independent. Following Carrol et al. (2018), this study investigated these variables through the lens of language users’ judgments and their ability to reliably comprehend a range of common English and translated idioms, and novel metaphors. The study recruited 143 people from various language groups. We show that familiarity is a powerful predictor of transparency based on the participants’ evaluations. In terms of inferring meaning accurately, the model with an interaction between transparency and familiarity was the best fit. The findings showed that accurately predicting the meaning resulted in a greater increase in the transparency and decomposability ratings. We examined how these variables interacted to allow individuals to infer meaning at various levels for known and novel figurative words. Valuable classroom time should be dedicated to teaching the least familiar, transparent, and decomposable figurative meanings of FPs. This can be accomplished by employing cognitive methods, such as metaphor awareness. In terms of the amount of time invested and expected learning outcomes, future research must focus on determining the most effective teaching method.

## Data availability statement

The raw data supporting the conclusions of this article will be made available by the authors, without undue reservation.

## Ethics statement

The studies involving human participants were reviewed and approved by Taif University. The patients/participants provided their written informed consent to participate in this study.

## Author contributions

The author confirms being the sole contributor of this work and has approved it for publication.
